# Sodium Dodecyl Sulfate–Mediated Graphene Sensor for Electrochemical Detection of the Antibiotic Drug: Ciprofloxacin

**DOI:** 10.3390/ma15227872

**Published:** 2022-11-08

**Authors:** Rakesh R. Sawkar, Mahesh M. Shanbhag, Suresh M. Tuwar, Kunal Mondal, Nagaraj P. Shetti

**Affiliations:** 1 Department of Chemistry, Karnatak Science College, Dharwad 580001, Karnataka, India; 2 Department of Chemistry, K.L.E. Institute of Technology, Hubballi 580027, Karnataka, India; 3 Idaho National Laboratory, Idaho Falls, ID 83415, USA; 4 Department of Civil & Environmental Engineering, Idaho State University, Pocatello, ID 83209, USA; 5 Department of Chemistry, School of Advanced Sciences, KLE Technological University, Vidyanagar, Hubballi 580031, Karnataka, India; 6 University Center for Research & Development (UCRD), Chandigarh University, Gharuan, Mohali 140413, Punjab, India

**Keywords:** ciprofloxacin, voltammetry, graphene, sodium dodecyl sulfate, detection limit

## Abstract

The present study involves detecting and determining CIP by a new electrochemical sensor based on graphene (Gr) in the presence of sodium dodecyl sulfate (SDS) employing voltammetric techniques. Surface morphology studies of the sensing material were analyzed using a scanning electron microscope (SEM) and atomic force microscope (AFM). In the electroanalysis of CIP at the developed electrode, an enhanced anodic peak response was recorded, suggesting the electro-oxidation of CIP at the electrode surface. Furthermore, we evaluated the impact of the electrolytic solution, scan rate, accumulation time, and concentration variation on the electrochemical behavior of CIP. The possible electrode mechanism was proposed based on the acquired experimental information. A concentration variation study was performed using differential pulse voltammetry (DPV) in the lower concentration range, and the fabricated electrode achieved a detection limit of 2.9 × 10^−8^ M. The proposed sensor detected CIP in pharmaceutical and biological samples. The findings displayed good recovery, with 93.8% for tablet analysis and 93.3% to 98.7% for urine analysis. The stability of a developed electrode was tested by inter- and intraday analysis.

## 1. Introduction

Fluoroquinolones (FQ) are a class of synthetic antibiotics frequently used to treat bacterial infections in humans and domestic animals. Ciprofloxacin (CIP) belongs to the third-generation FQ family of antibiotics. CIP is a broad-spectrum antimicrobial medication that inhibits DNA gyrase, a key enzyme in bacterial DNA replication, and has a bactericidal effect on Gram-positive and Gram-negative bacteria [1]. CIP medications are employed to prevent veterinary diseases in animals. It has a high propensity for absorption and circulation across fluids and tissues. This medicine treats various infectious disorders, including skin, urinary tract, respiratory, and gastrointestinal infections [2]. However, their widespread use may be responsible for pathogen resistance, including *Campylobacter*, *Salmonella*, and *E. coli* [3]. The adverse effects of CIP trigger swelling of a tendon, cardiac arrhythmia, agranulocytosis, TEN (toxic epidermal necrolysis), Stevens–Johnson syndrome, and thrombocytopenia, and overdose of CIP causes severe hepatotoxicity [4].

For the determination of CIP, wide ranges of analytical approaches have been employed, namely, HPLC [5,6,7], LC [8], spectrofluorometry [9], fluorometry [10], spectrophotometry [11], colorimetry, and capillary zone electrophoresis [12,13]. Since these traditional techniques are costly and time-consuming, they require a highly skilled technician to monitor, and some of the techniques are associated with complex procedures. However, electrochemical techniques can be an alternative method as they provide a better performance, robustness, and a faster reaction rate.

Electrochemical techniques have been widely used to determine various chemicals and biological components in multiple samples [14,15,16]. Electrochemical procedures are rapid, sensitive, and cost-efficient and can be miniaturized for portability [17,18]. Recently, nanomaterials-based electrochemical sensors have been advanced to investigate a variety of analyte molecules [19,20]. Carbon paste electrodes (CPE) are pivotal in determining analyte molecule electrodes such as glassy carbon electrodes (GCE) and carbon films. However, GCE has a small surface area, providing sluggish electron transfer [21]. Per reports, carbon film electrodes demand a very high temperature for preparation through pyrolysis [22,23]. On the other hand, the CPE drew the most attention as a working electrode in electrochemical analysis, providing high mechanical stability, increased sensitivity, selectivity, and stability [24,25]. The principal advantage of using CPE involves easy fabrication, cost efficiency, a broader potential window, low residual current, chemically inert, porous surface, low ohmic resistance, safe disposability, and ability to modify and refurbish the surface. Passivation issues are easily resolved throughout investigations by a rapid and simple surface regeneration [26,27,28,29]. CPEs are very well suited for organizing an electrode material that can be altered with mixtures of numerous other chemicals, giving the electrode some desired features. Such designed electrodes are susceptible sensors for sensing both inorganic and organic compounds. In addition, CPEs are environmentally friendly, nontoxic electrodes [30,31,32]. Metal nanoparticles, graphene, clay particles, dyes, polymers, and other nanomaterials can modify the CPE, resulting in reduced surface fouling, faster electron transport, and improved electrocatalysis [33,34,35,36]. As a result, these modifier materials were widely used to renovate the surface of bare CPEs to create a more perceptible surface [37,38].

Graphene (Gr) is a two-dimensional nanosheet formed via sp^2^ carbon hybridization in a hexagonal arrangement. Because of its unique electronic structure, two-dimensional character, flexibleness, and chemical stability, Gr has received much attention [39,40]. Gr has a broad array of applications in the field of nanodevices [41], corrosion [42], energy materials [43], and biomedical applications [44]. High surface area, remarkable thermal conducting power, and excellent binding capacity are characteristics of well-organized standard graphene sheets that qualify them as viable materials for various scientific fields [45]. Gr and its derivatives have been widely exploited in advancing biosensors for food preservation, environmental issues, and the biomedical symptomatic inquiry procedure [46,47]. Gr has significant benefits in biosensor fabrication as it has a wide surface area, excellent optical properties, extraordinary ability to interact with biomolecules, and good conducting properties [48].

Surfactants are amphiphilic surface-active agents widely employed in cleaning, developing paints and pigments, and manufacturing rubber and resins due to their distinctive molecular structure [49]. In electrochemical measurements involving voltammetric techniques, the surfactant’s incorporation on the electrode’s surface generates an adsorptive layer that could help in electron transfer, increasing the peak intensity and improving the redox potential [50]. Sodium dodecyl sulfate (SDS) is an anionic surfactant that can adjust and manage the electrode surface’s properties. It helps to increase the reaction rate and quickly accumulates at the electrode surface through the electrostatic force of attraction to promote the transfer of electrons between the electrode interface and analyte molecules. Hence, in the present investigation, SDS was added to increase the electrochemical responses of organic compounds and inorganic ions. Thus, they are very beneficiary in the field of electrochemistry. Many studies have been published on the various properties of surfactants in the field of electrochemistry [51,52].

The present research aims to establish a selective and sensitive electrode for determining CIP. Hence, this study involves a sodium dodecyl sulfate–mediated graphene-modified carbon paste electrode. The method involves easy fabrication of electrodes by preparing paste and packing inside a Teflon tube, followed by adding SDS to the test solution. On the other hand, some modification techniques, such as drop casting and electro-polymerization methods, require much time. The modification layer formed on the electrode surface can sometimes be a problem due to the thickness of films, which can alter voltammetric results, producing varied results. The main advantage of the present method is the ability to modify and refurbish the surface. Passivation issues are easily resolved throughout the investigations by a rapid and simple surface regeneration. Furthermore, electrode fabrication involves cheap, nontoxic, and environmentally friendly materials. We have investigated the electrochemical behavior of CIP at graphene-modified CPE in the presence of SDS (SDS·Gr/CPE). Voltammetric approaches such as cyclic voltammetry (CV) and differential pulse voltammetry (DPV) were utilized in the investigation.

## 2. Materials and Methods

### 2.1. Used Chemicals and Reagents

CIP (purity ≈ 99.9%) and Gr powder (purity ≥ 99.0%) were acquired from Sigma-Aldrich (Bangalore, India). SDS was obtained from HI Media Pvt. Ltd., Bangalore, India. All the other reagents utilized were of analytical quality. Phosphate buffer solution (PBS) of 0.2 M ionic strength was employed as an electrolyte solution, and PBS with varying pH (3.0 to 6.0) was formulated for the analysis.

### 2.2. Equipment and Instruments Used

An electrochemical workstation (CHI 1112C Model, CH Instruments, Inc., Austin, TX, USA) comprising a three-electrode system was assisted with a 10.0 mL voltammetric cell to investigate the electrochemical behavior of CIP using SDS·Gr/CPE as the working sensor. The saturated Ag/AgCl electrode is the standard reference, and the platinum wire is the auxiliary electrode. The solution pH of the formulated PBS was evaluated using a pH meter (Equiptronix, Bangalore, India). An atomic force microscope (AFM: Nanosurf, Liestal, Switzerland) and a scanning electron microscope (SEM: JEOL make, JSM-IT 500LA, Tokyo, Japan) were utilized to examine the topography and morphology of the modified electrode matrix.

### 2.3. Preparation of Standard Tablet and Urine Samples

The CIP tablets (CIPLOX 500) were obtained from the nearby pharmacy and were coarsely grounded using a mortar pestle. The appropriate amount of the powder form of the tablet was dissolved in double-distilled water to make the standard stock solution. The stock solution was subjected to sonication to attain complete dissolution. A known amount of a sample aliquot was then analyzed using the standard addition method, and recovery measurements were evaluated employing DPV.

The human urine samples were gathered from healthy individuals and were centrifuged at ambient temperature. The supernatant solution was then spiked with a predetermined quantity of CIP. The DPV technique was employed for the analysis with maintained optimized conditions. The content of the CIP in tablet and spiked urine samples was analyzed to determine the recovery at the proposed SDS·Gr/CPE.

### 2.4. Preparation and Evaluation of Fabricated Electrode

CPE was developed by homogeneous blending graphite and binder (paraffin) at 70:30 ratios. The obtained homogeneous paste was filled in a Teflon tube of 3 mm inner diameter and was polished to have a clean and shiny surface. An appropriate amount of Gr powder (0.05 g) was added along with graphite and binder to obtain a Gr/CPE. The SDS·Gr/CPE was developed by adding 1.0 mM of SDS (100.0 µL) into the electrolytic cell. After each measurement, the paste was removed from the Teflon tube and reloaded with a new paste, and SDS was added. The electrode prepared was washed and subjected to pretreatment before taking measurements.

To compute the active area of the Gr/CPE sensor, a CV approach was opted for, where K_3_[Fe(CN)_6_] of 1.0 mM concentration (C*) was used as a standard test solution in 0.1 M KCl solution. The CV measurements were measured for various scan rates (υ), and the Randles–Sevcik (1) equation was employed to compute the surface area (A) of the working sensor [53]. The slope values from the plot of Ip vs. υ^1/2^ for bare CPE and Gr/CPE were found to be 33.75 and 70.29 μA, respectively. The surface area of the sensors was calculated to be 0.045 and 0.095 cm^2^ for CPE and Gr/CPE, respectively:

I_p_ = (2.69 × 10^5^) n^3/2^Dₒ^1/2^ C*A ʋ^1/2^ (1)

where C is a concentration of K_3_[Fe(CN)_6_], that is, 1 × 10^−3^ M; n is the number of electrons transferred that equals 1; A is the active surface area of the electrode; and Dₒ is the diffusion coefficient with a value of 7.26 × 10^−6^ cm^2^ s^−1^.

## 3. Results and Discussions

### 3.1. Topography and Morphology Studies

**Figure 1:**
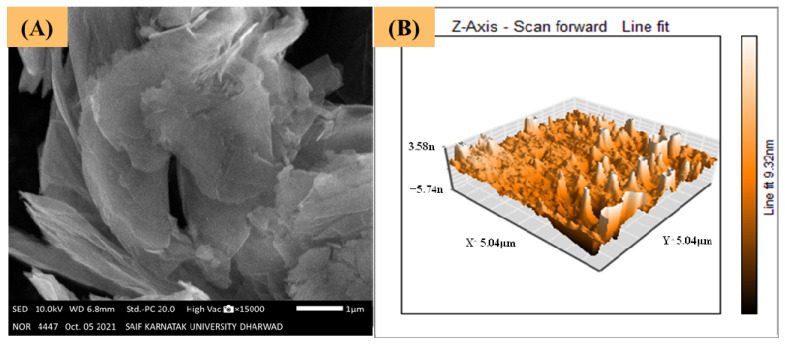
Surface topography and morphology of a modifier: (**A**) SEM image and (**B**) AFM image of the graphene matrix.

The surface topography and graphene morphology were studied using AFM and SEM analysis. The graphene’s SEM image (Figure 1A) shows a folded sheet randomly linked together. There are no amorphous phase particles in the sample, and they possess a sheetlike form. The flaky texture of Gr indicates its layered nanostructure, which is reflected in its morphology. By employing the AFM technique, the roughness of the surface of the proposed sensor can be determined and was found to be 25.59 pm^2^. The increased roughness of the sensor aids in improving the sensor’s sensitivity, implying that the sensor has been effectively modified, which plays a significant part in electrochemical determination. The AFM image of the graphene matrix is shown in Figure 1B. The presence of a single peak at 2ϴ° = 26.48° confirms the excellent purity of graphene with a microstructure particle size (Figure S1).

### 3.2. Impact of Electrode Modification

The choice of an electrode is essential in the electrochemical detection of an analyte molecule. Initially, the electrochemical oxidation of CIP was investigated at bare GCE and bare CPE. At both electrodes, a single oxidation peak was noticed for 0.1 mM CIP in pH 4.2 PBS. However, the peak obtained at CPE was highly intense and sharp compared with GCE (Figure S2A), which verifies the effectiveness of CPE.

To determine the charge transfer capacity of the developed electrodes, electrochemical impedance spectroscopy (EIS) assay was performed in 1.0 mM K_3_[Fe(CN)_6_] in 0.1 M KCl (as electrolyte). The EIS was recorded at 0.429 V, and the Nyquist plot for the electrodes is given in Figure S2B. The charge transfer resistance values were found to be 83.09, 53.45, and 35.01 kΩ for CPE, Gr/CPE, and SDS·Gr/CPE, respectively. The obtained values indicate poor and sluggish charge transfer, suggesting lower conductivity, while SDS·Gr/CPE shows lower charge transfer resistance, indicating higher charge transfer efficiency with good conductivity.

**Figure 2:**
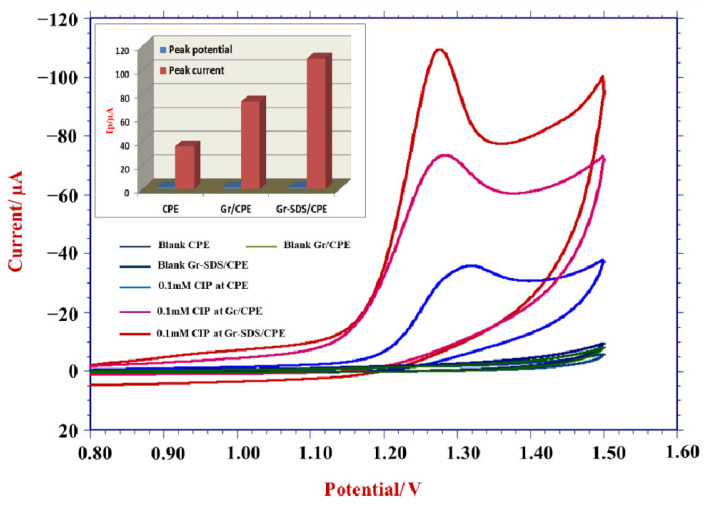
Electrochemical nature of CIP (0.1 mM) at different electrodes at pH 4.2 of phosphate buffer solution (PBS) at a scan rate of 0.05 Vs^−1^.

Further, the CV responses of CIP at CPE, Gr/CPE, and SDS·Gr/CPE were recorded at 0.05 Vs^−1^ in pH 4.2 (Figure 2). No peaks were observed in the absence of CIP. However, a single oxidation peak of CIP was detected at all three electrodes, and the irreversible nature of the system was confirmed by the absence of a peak in the reverse scan [54]. The CV of bare CPE displayed an anodic peak current at 35.85 µA at 1.318 V, while at Gr/CPE, the peak was detected at 1.284 V with a peak current of 73.4 µA. Further, for SDS·Gr/CPE, a well-oriented peak was obtained with a peak current of 108.9 µA at a potential of 1.275 V. Moreover, better augmentation in peak intensity and peak potential was obtained at SDS·Gr/CPE compared |with bare CPE and Gr/CPE.

### 3.3. Accumulation Time

**Figure 3:**
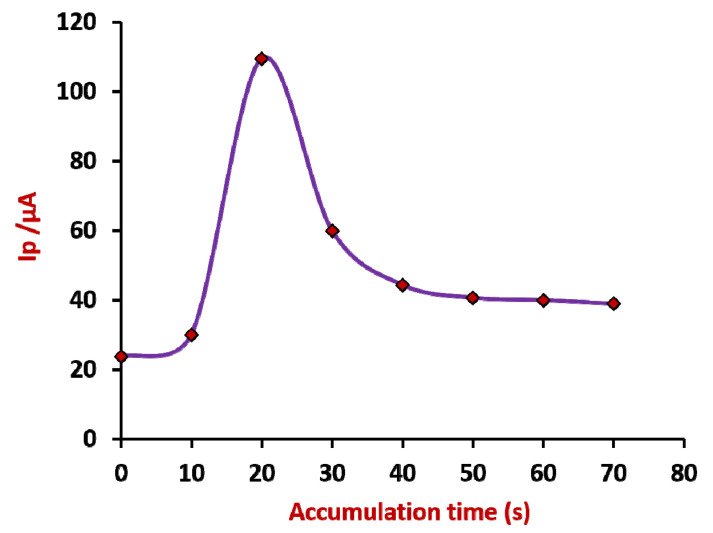
Graphical representation of accumulation time of CIP at pH 4.2 (PBS) at SDS·Gr/CPE.

In voltammetric measurements, the electrochemical reaction of the analyte molecule at the sensor’s surface depends on the interaction duration between the analyte molecule and the electrode surface. That suggests that the analyte concentration at the sensor vicinity could affect the electrochemical reaction. Hence, the accumulation time effect was investigated using the CV approach for 0.1 mM CIP within the time interval from 0 to 70 s. It can be visualized from Figure 3 that the peak current increased gradually from 0 to 20 s, and after 20 s, the current declined, and steady voltammograms were obtained with a lower peak current value. However, we noticed a well-oriented and intensified peak at 20 s due to the high concentration of CIP molecules aggregating near the electrode surface, which causes the peak current to increase and directly affects the electrode’s sensitivity. Further, after 20 s of accumulation time, the saturation limit was reached, and the peak current decreased and maintained a steady range. As the maximum peak intensity was observed for 20 s of accumulation time, it was optimum to pursue further investigations.

### 3.4. Impact of an Electrolytic Solution

**Figure 4:**
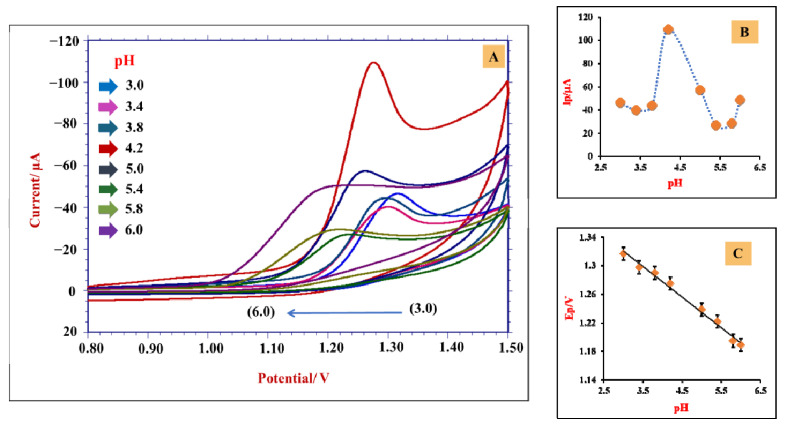
(**A**) Cyclic voltammograms depicting the impact of pH (3.0–6.0) on the oxidation of CIP at SDS·Gr/CPE at a 0.05 Vs^−1^ effective scan rate. (**B**) Dependency of a peak current on pH. (**C**) Dependency of a peak potential on pH.

The investigation of the electrochemical behavior of a specific analyte molecule at different pH conditions has a substantial role in understanding the reaction mechanism. Thus, the influence of supporting buffer (0.2 M PBS) on the electro-oxidation of CIP at modified CPE was investigated by varying pH (3.0–6.0) using the CV technique at 0.05 Vs^−1^ (Figure 4A). It was noticed that the peak potential switched towards a less positive potential as the pH of the PBS solution was raised, implying that protons participated in the reaction [55]. Further, the maximum peak current was detected for PBS of pH 4.2 (Figure 4B), suggesting that pH variation impacts peak current, and hence, pH 4.2 has opted for further analysis. The plot of E_p_ vs. pH is presented in Figure 4C with a linear equation: E_p_ = −0.0427 pH + 1.448; R^2^ = 0.989.

The obtained slope value (0.042) was near the Nernstian value (0.059), implying the participation of an equal number of electrons and protons during the reaction [56].

### 3.5. Influence of Scan Rate

**Figure 5:**
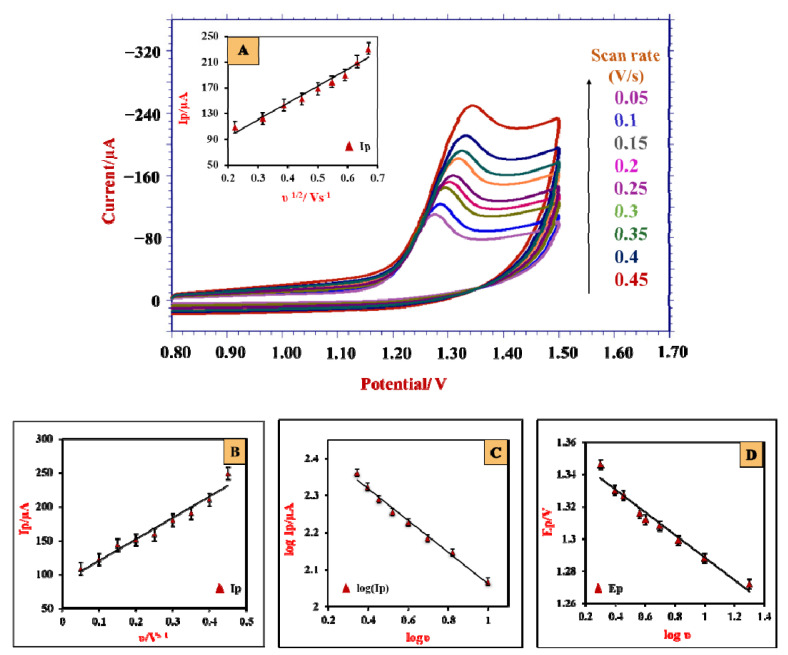
Cyclic voltammograms for 0.1 mM CIP in pH 4.2 at varied scan rates ranging from 0.05 to 0.45 Vs^−1^. (**A**) plot of I_p_ vs. υ^1/2^, (**B**) plot of I_p_ vs. υ, (**C**) plot of logI_p_ vs. logυ, and (**D**) plot of Ep vs. logυ.

The impact of scan rate variation provides physicochemical information on the reaction mechanism and electrode process. An electrochemical oxidation of 0.1 mM CIP was investigated using pH 4.2 at modified CPE for different scan rates employing the CV approach. It can be noticed from Figure 5 that the peak current was raised gradually with a rise in scan rate since the increase in scan rate leads to a decrease in the size of the diffusion layer. A linear relationship was observed for the plot of I_p_ vs. υ^1/2^ with the regression equation I_p_ = 263.61υ^1/2^ + 41.28; R^2^ = 0.969 (Figure 5A). The plot of Ip vs. υ gave the linear equation I_p_ = 316.7υ + 89.04; R^2^ = 0.960 (Figure 5B), which suggests the surface-confined process. Further, the plot obtained for logI_p_ vs. logυ (Figure 5C) displayed the linear curve with the fitted equation logI_p_ = 0.428logυ + 2.491; R^2^ = 0.998. However, the slope value of 0.42 verifies that diffusion controls the process [57,58].

Furthermore, good linearity was observed for the plot of E_p_ vs. logυ, which can be observed in Figure 5D, and the corresponding regression equation is as follows:

E_p_ = 0.0701logυ + 1.358; R^2^ = 0.968

For the system comprising irreversible reaction, Laviron’s equation [58] can be given as:

Ep = E^0^ + [2.303RT/(1 − α) nF] · log[(1 − α) nF/RTk^0^] + [2.303RT/(1 − α)nF] logʋ (2)

where E^0^ is formal redox potential, α refers to the transfer coefficient, k^0^ refers to the rate constant, and the remaining notations denote their standard meanings. By calculation, the electrons transferred in the reaction and the rate constant were found to be 1.68 ≈ 2 and 2.01 s^−1^, respectively.

### 3.6. Plausible Electro-Oxidation Reaction of CIP

**Scheme 1:**
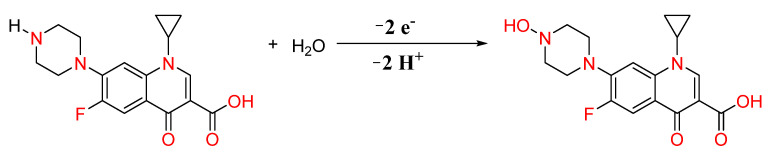
Electrochemical oxidation reaction of CIP.

The electrochemical oxidation mechanism involved the participation of an even number of protons and electrons, which was validated by pH studies. Furthermore, scan rate investigations found the electron number to be 2. These speculations show the possible reaction mechanism of CIP at SDS·Gr/CPE in Scheme 1 [59]. CIP has a secondary amine group that forms a primary center with a lone pair of electrons as a donor. On this basis, the reaction occurs through the loss of two protons and two electrons, which proceeds through the oxidation of secondary amine (-NH), the only active site available for the oxidation to give an N-hydroxylation derivative. The -NH- group of CIP is oxidized to the -N-OH group with the loss of two protons and two electrons.

## 4. Analytical Applications

### 4.1. Quantification of CIP

**Figure 6:**
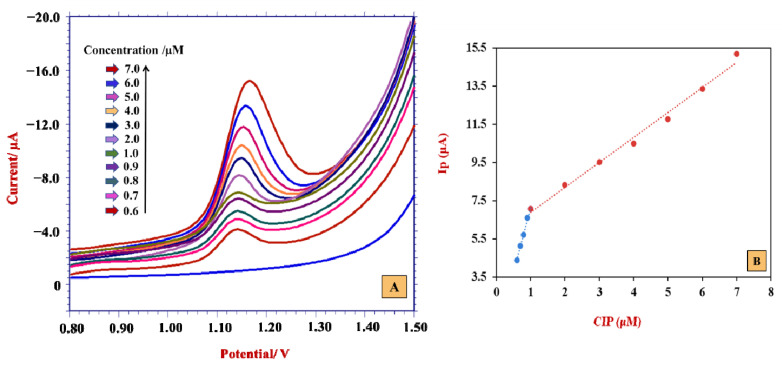
(**A**) Differential pulse voltammograms depicting variation in CIP concentrations ranging from 0.6 to 7 µM using PBS of pH 4.2 at SDS·Gr/CPE. (**B**) Dependence of I_p_ on concentration (at a lower concentration).

Concentration variation studies of CIP at SDS·Gr/CPE were performed using DPV at pH 4.2 (PBS). In addition, DPV is known for its sensitivity; hence, the DPV technique has opted for concentration studies. Figure 6A shows the DPV responses for various concentration ranges of CIP from 0.6 to 7 µM. The increase in concentration leads the peak current to increase. We obtained linearity for the concentration mentioned above, and the linearity range for lower concentrations was selected (Figure 6B) to validate the sensor’s quantification. The regression equation for the calibration curve was obtained as I_p_ = 6.92C + 0.232; R^2^ = 0.992. The detection limit (L_D_) and quantification limit (L_Q_) values were determined using the equations below [60], and the calculated L_D_ and L_Q_ were found to be 0.03 and 0.1 µM, respectively:

L_D_ = 3 × s/m (3)

L_Q_ = 10 × s/m (4)

where s corresponds to the standard deviation of the intercept, and m represents the slope. The characteristics of the concentration variation plot are described in Table 1. The L_D_ and L_Q_ values were lower than other reported works from the literature (Table 2). The lower L_D_ value shows that the fabricated sensor is suitable for trace-level detection of CIP. In addition, the method is cost-efficient, the electrode fabrication is easy and time-saving, the development of a sensing surface is easy, unlike the drop-casting and polymerization method, which saves time. Furthermore, the electrode developed can provide reproducible results and the lowest L_D_ value compared with some of the methods listed in Table 2.

### 4.2. Impact of Interferers

**Figure 7:**
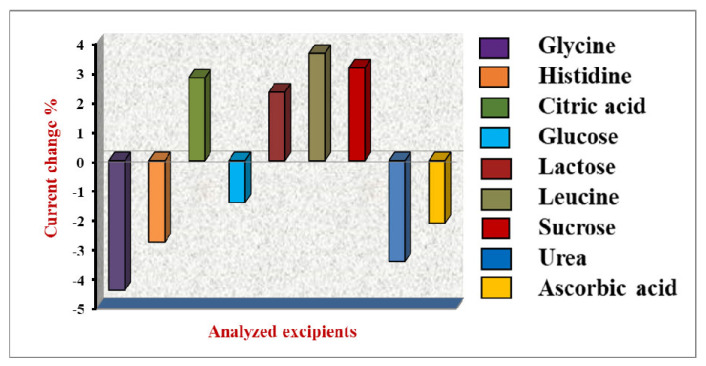
Effect of excipients on the peak current of CIP.

Some of the most used biological metabolites (excipients) were employed to assess the interference in the electrochemical process of CIP. The investigation was carried out for 3.0 µM CIP and 10-fold excess addition of excipient samples along with the addition of SDS (100.0 µL) in the test solution. The outcome of this investigation shows that the potential peak value and peak current values have been changed to some extent, but they did not exceed the ±5% limit. This suggests that these excipients do not affect the CIP detection at SDS·Gr/CPE. Figure 7 shows the % change of the peak current, and Table S1 describes the experimental findings of the excipient analysis. 

### 4.3. Recovery Studies

The developed sensor was evaluated for determining CIP in pharmaceutical samples employing the DPV technique at pH 4.2 of PBS. The preparation of tablet samples is outlined in Section 2.3. The experimental findings are displayed in Table 3. The results revealed a good recovery of 93.88% and good agreement with the manufacturer’s labeled content.

In addition, the sensor was utilized to determine CIP in spiked urine samples. The test samples for the analysis were prepared according to the procedures outlined in the experimental Section 2.3. A pH 4.2 buffer solution was used to dilute the samples. The biological fluid sample was ready for analysis by adding a known volume of analyte solutions to the urine samples. For recovery measurements, the DPV method was exploited. The results displayed an excellent retrieval range of 93.83–97.82% (Table 4).

### 4.4. Stability of SDS·Gr/CPE

The proposed sensor’s repeatability was tested using 1.0 µM CIP. The sensor was kept in a sealed jar for about 15 days and was subjected to taking measurements, and the sensor preserved its prior peak current response with 97.4% recovery. The voltammograms showing repeatability investigations are displayed in Figure S3A, demonstrating that the prepared electrode is stable over time.

The developed sensor was employed for intraday trials at a constant temperature to investigate the electrode’s reproducibility. Five subsequent measurements were taken for 1.0 µM of CIP by maintaining constant concentration. The % RSD value found to be 3.19 indicates that the sensor possesses good reproducibility for CIP detection. The concerned data are provided in Figure S3B. These findings suggest that the designed sensor material has excellent stability and repeatability.

## 5. Conclusions

A simple, rapid, and cost-effective electrochemical sensor was developed employing Gr for detecting a trace amount of CIP. The surface morphology and topography of the sensor were evaluated using the SEM and AFM approaches. The electrochemical characterization of the developed sensor was carried out using CV and EIS studies. The electrochemical analysis of CIP was carried out utilizing the Gr/CPE sensor in the presence of SDS at its optimum pH of 4.2 (PBS), employing the voltammetric technique. A higher active surface area and a low charge resistance at Gr/CPE help sensitively detect CIP. Electrochemical investigations showed an increased peak current for CIP at SDS·Gr/CPE compared with nascent CPE. The data revealed that diffusion phenomena controlled the process by participating in two electrons and two protons. The possible electrode mechanism was predicted and proposed with the acquired information. For the developed sensor, good linearity was observed, and a lower L_D_ of 0.029 µM was achieved. Furthermore, the determination of CIP in tablet and urine samples demonstrated the applicability of the developed sensor, while interference illustrated the selectivity. The results indicated that the method is feasible, rapid, reliable, and useful for determining CIP, even in the presence of excipients. 

## Figures and Tables

**Table 1:** Calibration features of CIP.

Calibration Characteristics	CIP
Linearity range	0.3 × 10^−6^ M to 100.0 × 10^−6^ M
Concentration range	0.6 × 10^−6^ M to 1.0 × 10^−6^ M
Value of slope	6.92
Standard deviation of slope	0.083
Value of intercept	0.232
Standard deviation of intercept	0.067
Regression coefficient	0.992
Total data points	05
L_D_	0.03 μM
L_Q_	0.1 μM

**Table 2:** Comparative study of detection limits with other reported works.

Electrode	L_D_ (µM)	L_Q_ (µM)	Linear Range (μM)	pH	Reference
GNs-GCE (DPV)	0.02	-	0.5–200	2.0	[59]
Carbon nanospheres (DPV)	0.15	-	0.5–5	7.0	[61]
Cd(II)/graphene-based electrode (ASV)	0.05	-	0.1–10	3.6	[62]
Surfactant-CPE (DPV)	0.18	0.61	2.0–4.0	6.5	[63]
Thiol-grafted laponite GCE (DPV)	0.26	-	10–110	2.0	[64]
SDS·Gr/CPE (DPV)	0.029	0.096	0.3–100	4.2	Present method

GCE—glassy carbon electrode, GNs—graphene nanoparticle, CPE—carbon paste electrode, SDS·Gr/CPE—sodium dodecyl sulfate–mediated graphene-modified carbon paste electrode, DPV—differential pulse voltammetry, ASV—anodic stripping voltammetry.

**Table 3:** Detection of CIP in tablet formulations.

Mentioned amount (mg)	500
Amount obtained (mg) ^a^	471
RSD (%)	3.04
Added (mg)	1.0
Obtained (mg) ^a^	0.93
Recovered (%)	93.8

^a^ Average of three measurements.

**Table 4:** CIP detection in urine samples.

Sample No.	Spiked (×10^−5^ M)	Obtained (×10^−5^ M)	Recovery (%)
#1	0.1	0.09	98.7
#2	0.2	0.19	95.0
#3	0.3	0.28	93.3

## Data Availability

Not applicable.

## Supplementary Materials

The following supporting information can be downloaded at: https://www.mdpi.com/article/10.3390/ma15227872/s1, Figure S1: XRD image of graphene. Figure S2: Voltammetric response of 0.1 mM CIP in PBS of 4.2 pH using bare CPE and bare GCE at a scan rate of 0.05 V/s (A); EIS study of developed electrodes—Nyquist plot (B). Figure S3: Voltammograms obtained for repeatability studies (A); reproducibility studies (B). Table S1: Data obtained for excipient analysis.
