# Supplementary material for: Sodium Dodecyl Sulfate–Mediated Graphene Sensor for Electrochemical Detection of the Antibiotic Drug: Ciprofloxacin
Source: Materials (Basel). 2022 Nov 8;15(22):7872. doi: 10.3390/ma15227872 (PMC9696905; doi:10.3390/ma15227872)
Supplement: Supplementary file 1 [file materials-15-07872-s001.zip › materials-1949278-supplementary.pdf]

Article

Sodium Dodecyl Sulfate Mediated Graphene Sensor for Electrochemical Detection of the Antibiotic Drug: Ciprofloxacin

Rakesh R. Sawkar [1], Mahesh M. Shanbhag [2], Suresh M. Tuwar [1], Kunal Mondal [3,4,*] and Nagaraj P. Shetti [5,6,*]

Supplementary information

Figures:

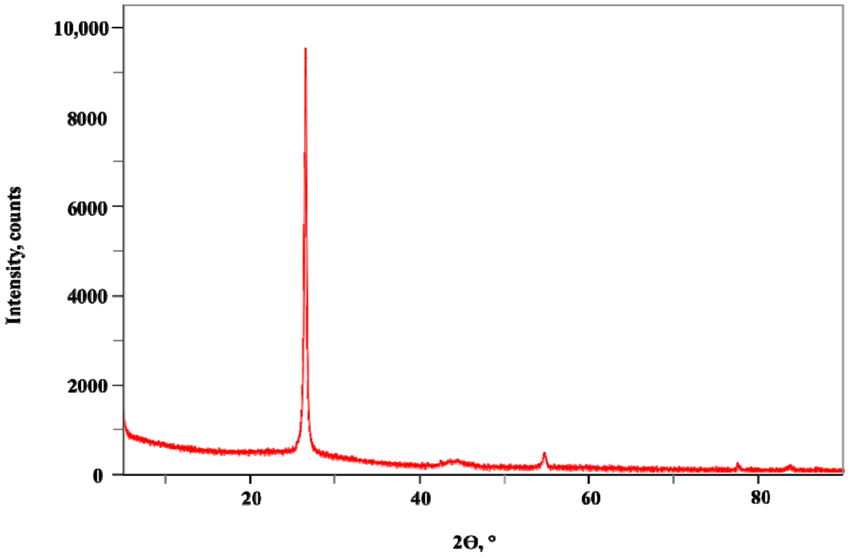

Figure S1. XRD image of graphene.

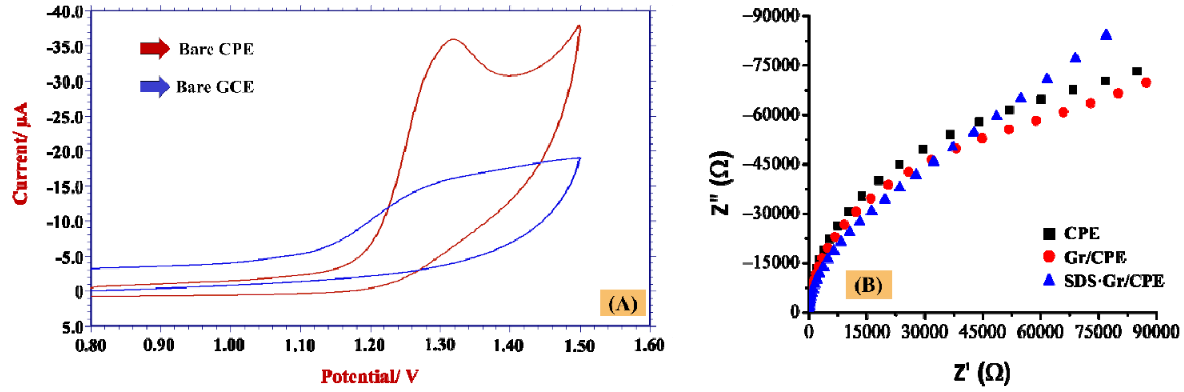

Figure S2. Voltammetric response of 0.1mM CIP in PBS of 4.2 pH using bare CPE and bare GCE at a scan rate of 0.05V/s **(A)**; EIS study of developed electrodes – Nyquist plot **(B)**.

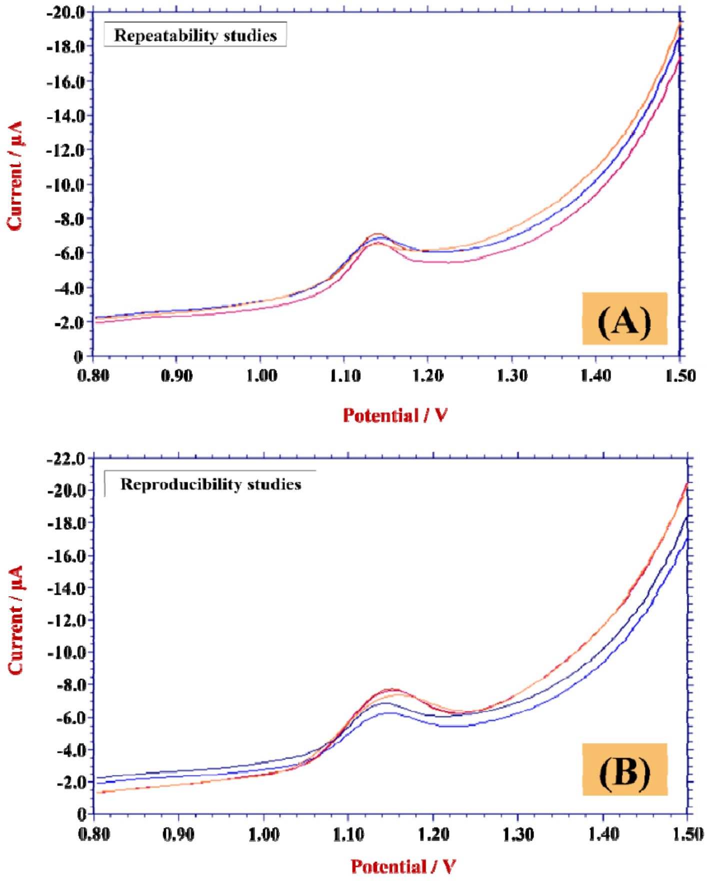

Figure S3. Voltammograms obtained for repeatability studies **(A)**; Reproducibility studies **(B).**

Tables:

Table S1. Data obtained for excipient analysis

Analysed excipients	Current observed *	Signal Change % (Ip)
CIP	9.55	-
Glycine	9.98	-4.41
Histidine	9.82	-2.77
Citric acid	9.28	2.81
Glucose	9.69	-1.43
Lactose	9.33	2.33
Leucine	9.20	3.65
Sucrose	9.25	3.15
Urea	9.88	-3.43
Ascorbic acid	9.76	-2.13

* Average of three readings
